# TMPRSS2 Correlated With Immune Infiltration Serves as a Prognostic Biomarker in Prostatic Adenocarcinoma: Implication for the COVID-2019

**DOI:** 10.3389/fgene.2020.575770

**Published:** 2020-09-30

**Authors:** Lianxiang Luo, Yushi Zheng, Mingyue Li, Xinjie Lin, Xiaodi Li, Xiaoling Li, Liao Cui, Hui Luo

**Affiliations:** ^1^ The Marine Biomedical Research Institute, Guangdong Medical University, Zhanjiang, China; ^2^ The Marine Biomedical Research Institute of Guangdong Zhanjiang, Zhanjiang, China; ^3^ The First Clinical College, Guangdong Medical University, Zhanjiang, China; ^4^ Department of Pathology and Laboratory Medicine, Perelman School of Medicine, University of Pennsylvania, Philadelphia, PA, United States; ^5^ Animal Experiment Center, Guangdong Medical University, Zhanjiang, China; ^6^ Guangdong Key Laboratory for Research and Development of Natural Drugs, Guangdong Medical University, Zhanjiang, China

**Keywords:** type 2 transmembrane serine protease, coronavirus disease 2019, prostatic adenocarcinoma, immune infiltration, prognostic biomarker

## Abstract

Type 2 transmembrane serine protease (TMPRSS2) is a new member of the serine proteases, and studies have shown that TMPRSS2 plays a role in the occurrence of prostate malignancies and is closely related to the occurrence of the coronavirus disease 2019 (COVID-19). However, the role of TMPRSS2 in prostatic adenocarcinoma (PRAD) remains largely unclear. To better explore its function in PRAD, we examined the expression level of TMPRSS2 in the GEO, tumor immune assessment resource (TIMER), as well as Oncomine databases and studied the association between TMPRSS2 and overall survival (OS) rates in the UALCAN and gene expression profiling interactive analysis (GEPIA) databases. In addition, we studied the correlation of the level of immune infiltration and markers of immune cell type in the TIMER database, analyzed the prognosis based on the expression level of TMPRSS2 in the related immune cell subsets, and determined the methylation profile of TMPRSS2 promoter by UALCAN database. Subsequently, we conducted a survival analysis and gene ontology (GO) pathway analysis in the TISID database and detected the expression of TMPRSS2 in the Human Protein Atlas (HPA) database. We also studied the protein-protein interaction (PPI) network of TMPRSS2 in the GENEMANIA database. Additionally, we used the microarray GSE56677 and GSE52920 to illustrate changes in TMPRSS2 expression *in vivo* and *in vitro* after severe acute respiratory syndrome-coronavirus (SARS-COV) infection, finding that expression of TMPRSS2 decreased after SARS-COV infection *in vitro*. The function of TMPRSS2 in the dataset was further verified by gene set enrichment analysis (GSEA). In conclusion, the expression of TMPRSS2 is significantly increased in PRAD, elevated TMPRSS2 is associated with immune infiltration, and prognosis is positively correlated. In addition, tumor tissue from COVID-19 patients with PRAD may be more susceptible to infection with SARS-COV-2, which may render the prognosis gets worse.

## Introduction

Prostatic adenocarcinoma (PRAD) is one of the most common causes of cancer-related death in men in the United States (Gupta et al., 2000). Prostate cancer is one of the leading causes of morbidity and mortality in men 50 years of age, whose incidence rate varies in different countries and ethnic groups (Parsons et al., 2001). In 2012, the incidence of prostate cancer in the tumor registration areas of China was 9.92/100,000, ranking sixth in male malignant tumors. The prostate consists of two components: the epithelium and the stroma. The interaction between the epithelial cells and the stroma is a key factor in the maintenance of normal function and homeostasis of the prostate (Cunha, 2008). In the past, treatment of advanced PRAD was limited due to the lack of effective drugs (Pchejetski et al., 2005). Therefore, before developing specific drugs, exploring the occurrence mechanism and identifying new tumor biomarkers with high sensitivity and specificity are crucial in addressing PRAD.

Recent studies have shown that TMPRSS2 is a new member of the serine proteases and has been reported to be associated with the intestine (Paolonigiacobino et al., 1997). At the same time, researchers later found that the gene was expressed primarily in the prostate in an androgen-dependent manner. The androgen-regulated TMPRSS2 promoters form a fusion gene with coding regions of the proto-oncogenic ETS transcription factor family members, which are closely related to prostate cancer and regulate many biological processes (Tomlins et al., 2005). Additionally, TMPRSS2 encodes an intracellular type II transmembrane protein, LDL receptor A (LDLRA), and the scavenger receptor cysteine-rich (SRCR) and serine protease domains (Vaarala et al., 2001). Because it is located on the surface of prostate cells, we found that TMPRSS2 could be a potential marker for prostate cancer diagnosis. However, the prognosis and immune mechanisms of TMPRSS2 in PRAD are still unclear.

In late December 2019, a case of viral pneumonia caused by a novel coronavirus was reported in Wuhan, Hubei province, China (Lu et al., 2020). The virus was referred to as severe acute respiratory syndrome-coronavirus (SARS-COV), which is an enveloped RNA virus that can cause intestinal, respiratory, and central nervous system diseases in a variety of animals and humans (Denison et al., 2011). As of August 28, 2020, more than 24.2 million confirmed cases have been reported across more than 200 countries and territories, resulting over 820,000 deaths (according to *data from Johns Hopkins University*) and causing a notable negative impact on human health and economic development. This coronavirus has been recognized by the World Health Organization as a public health emergency of international concern. Currently, no specific antiviral drugs or clinically effective vaccines are available to prevent and treat the coronavirus disease 2019 (COVID-19; Sanders et al., 2020). Several reports (mainly case series) from around the world have concluded that patients with malignant tumors seem to be more vulnerable to severe COVID-19 infection and death (Addeo and Friedlaender, 2020; Van De Haar et al., 2020), especially those with precancerous conditions (Bhowmick et al., 2020). However, the prognosis of COVID-19 patients with PRAD is unclear. Angiotensin-converting enzyme 2 (ACE2) has identified as cell entry receptors for SARS-COV-2, and receptor-mediated virus entry was dependent on TMPRSS2 (Hoffmann et al., 2020; Zhou et al., 2020). Studies have shown that TMPRSS2 can reduce viral response and promote viral transmission and pathogenesis (Glowacka et al., 2011). More specifically, TMPRSS2 can cleave the SARS-COV-2 spike protein, facilitating viral entry and activation (Strope et al., 2020), and TMPRSS2-expressing cell lines are highly susceptible to SARS-COV, Middle East respiratory syndrome-coronavirus (MERS-COV), and SARS-COV-2 (Matsuyama et al., 2020), which prompted us to explore the association between TMPRSS2 and SARS-COV-2, especially in PRAD patients.

In this work, we studied the mRNA expression level, overall survival (OS), and correlation with immune cells, among other factors. We used tumor immune assessment resource (TIMER), Oncomine database, and GTEx project to obtain the mRNA expression level of TMPRSS2 in PRAD. The prognostic value and OS rate of TMPRSS2 in PRAD were analyzed *via* the gene expression profiling interactive analysis (GEPIA) and UALCAN databases to explore its functional mechanism. Subsequently, we studied the correlation among TMPRSS2, immune infiltration level, and immune cell type markers in different tumors in the TIMER database. In addition, the integrated repository portal for tumor-immune system interactions (TISIDB) database was used in survival analysis and gene ontology (GO) pathway analysis, and we visualized the Protein-protein interaction (PPI) network in the GENEMANIA database. The expression of TMPRSS2 was detected in the Human Protein Atlas (HPA) database. In addition, GSE56677 and GSE52920 were used to study the expression changes of TMPRSS2 *in vivo* and *in vitro* after SARS-COV infection. Based on these data, we identified and elucidated the important role of TMPRSS2 in PRAD and the underlying mechanisms associated with its immune infiltration. The sensitivity of the tumor to SARS-COV-2 and the prognosis of PRAD in patients with COVID-19 were also illustrated.

## Materials and Methods

### Oncomine Database Analysis

The Oncomine database^1^ is a web-based data mining platform with a microarray database of most human cancers (Rhodes et al., 2007). In this study, the expression level of TMPRSS2 in PRAD was analyzed using the oncology database. In this study, we conducted a search based on the following criteria: (A) analysis type: cancer and normal tissue; (B) data type: mRNA; and (C) threshold: fold change = 1.5 and value of *p* = 0.01.

### TIMER Database Analysis

Using the TIMER database,^2^ this study analyzed the expression of TMPRSS2 in PRAD patients and six types of infiltration of immune cells (B-cells, CD4^+^ T-cells, CD8^+^ T-cells, neutrophils, macrophages, and dendritic cells) using an abundance of correlation (Li et al., 2017). At the same time, the correlation between TMPRSS2 expression and the genetic markers of tumor infiltrating immune cells was also discussed.

### GEPIA Database Analysis

In this study, interaction analysis was conducted on online database GEPIA^3^ to study the expression of PRAD based genes. Logrank inspection and Mantel-Cox test were used to generate the survival curve, including OS and relapse-free survival (RFS). GEPIA is an interactive network consisting of 9,736 tumor samples and 8,587 normal samples from the TCGA and GTEx projects that analyzed RNA sequencing expression (Tang et al., 2017).

### UALCAN Database Analysis

In this study, clinical data from TCGA3RNA-seq in UALCAN^4^ and clinical data from 31 cancer types were used to analyze the characteristics of tumor and normal samples in a single other clinic pathological stage, as well as the relative expression of different genes in the tumor subgroup (Chandrashekar et al., 2017).

### GENEMANIA Database Analysis

GENEMANIA^5^ is a network interface for hypothesis deduction based on gene function (Wardefarley et al., 2010). GENEMANIA can generate a list of genes with similar functions as a query and build an interactive functional association network to illustrate the relationship between genes and data sets. Using this database, we constructed the gene interaction network of TMPRSS2 for coexpression, colocalization and genetic interaction and systematically evaluated its function.

### Human Protein Atlas Database Analysis

The HPA^6^ was a program with the aim to map all the human proteins in cells, tissues, and organs using an integration of various Omics technologies (Uhlén et al., 2015; Uhlen et al., 2017), and it supplies 32 human tissues and their protein expression profiles and uses antibody analysis to accurately assess protein localization (Lanczky et al., 2016). In this study, we used HPA database to analyze the protein expression and immunohistochemistry (IHC) of TMPRSS2 in normal tissues and PRAD tissues.

### TISIDB Database Analysis

TISIDB database^7^ was used to further investigate the correlation between TMPRSS2 expression and lymphocytes and immune modulators. The TISIDB database, known as a portal for interaction between the tumor and immune systems, integrates 988 reported immune-related anti-tumor genes, high-throughput screening techniques, molecular profiling and paracancer multinomics data, and various immunological data resources retrieved from seven public databases (Ru et al., 2019).

### Microarray Data Collection

We obtained the SARS-COV-related microarray, GSE30589 (Dediego et al., 2011), GSE56677 (Selinger et al., 2014), and GSE52920 (Jimenez-Guardeno et al., 2014) expression profiles and the prostatic-related microarray GSE6956 (Wallace et al., 2008) in the GEO database,^8^ a microarray form of high-throughput functional genomics data for public knowledge base storage. The data were normalized *via* the limma package (Smyth et al., 2005) using the R language. This study elucidated the changes of TMPRSS2 in cells and animals infected with SARS-COV and found the important role of TMPRSS2 in PRAD patients.

### GTEx Database Analysis

GTEx database^9^ is a database that supplies tissue RNA-Seq data and SNP information contributed by healthy people and combines SNP information and gene expression level. This database was used to investigate the gene expression of TMPRSS2 in the prostate gland (Lonsdale et al., 2013).

### GO and KEGG Functional Enrichment Analysis

To explore the relevant pathways and functional annotation involved in TMPRSS2 in GSE30589 and GSE52920, we also conducted GO and Kyoto Encyclopedia of Genes and Genomes (KEGG) enrichment analyses. GO and KEGG analyses and visualization were implemented based on R software (Version 3.6.1). The results with *p* < 0.05 were selected.

### Gene Set Enrichment Analysis

A computational method known as gene set enrichment analysis (GSEA) was used to analyze the signature gene function and potential pathway. GSEA^10^ is a computational method that determines whether an *a priori* defined set of genes shows statistically significant and concordant differences between two biological states (20; e.g., phenotypes; from the official GSEA website). To explore another link between TMPRSS2 and the functions of interest and to enhance our understanding of the correlation between biological events, we used GSEA software version 4.0.3 and single-gene GSEA of two groups of GSEs based on “C5: GO sets” and “C7: Immunologic Gene Sets.” We set the cut-off criterion to a false discovery rate (FDR) < 25% and nominal *p* < 0.05.

## Results

### TMPRSS2 High Expression Level in Tumors

**Figure 1:**
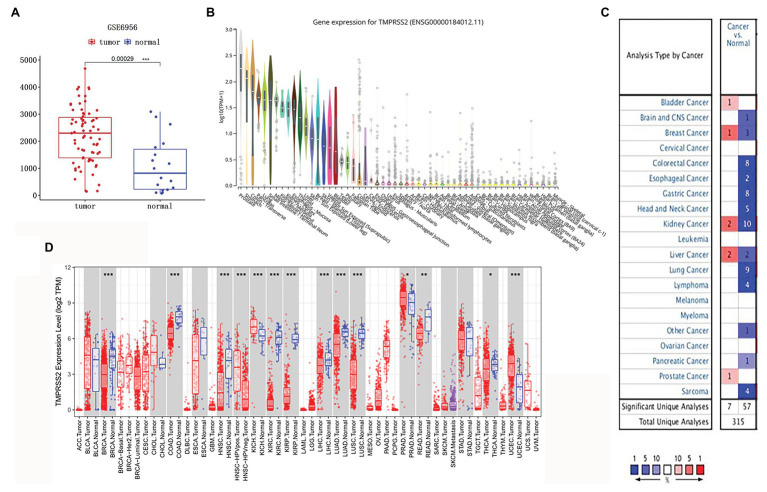
Type 2 transmembrane serine protease (TMPRSS2) expression level. **(A)** mRNA expression of TMPRSS2 in GEO. mRNA expression of TMPRSS2 is higher in tumor tissue but lower in normal tissue based on GEO samples; **(B)** Gene expression for TMPRSS2 in human organs. Gene expression of TMPRSS2 in the prostate is higher than in any other organ; and **(C)** mRNA expression level of TMPRSS2 in various cancers. Color images are available online. Fold change = 1.5, value of *p* = 0.01, and a top 10% of gene rankings; and **(D)** different TMPRSS2 expressions between tumor and adjacent normal tissues. ^*^ *p* < 0.05, ^**^ *p* < 0.01, and ^***^ *p* < 0.001.

**Figure 2:**
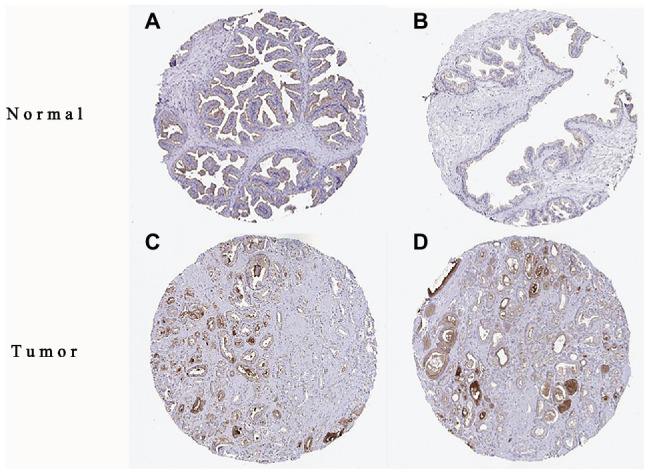
Immunohistochemistry (IHC) of TMPRSS2 expression in prostatic adenocarcinoma (PRAD) tissues and corresponding normal tissues based on the Human Protein Atlas (HPA). **(A,B)** Normal prostate (T-77100) tissue; **(C,D)** prostate (T-77100) tumor tissue.

**Figure 3:**
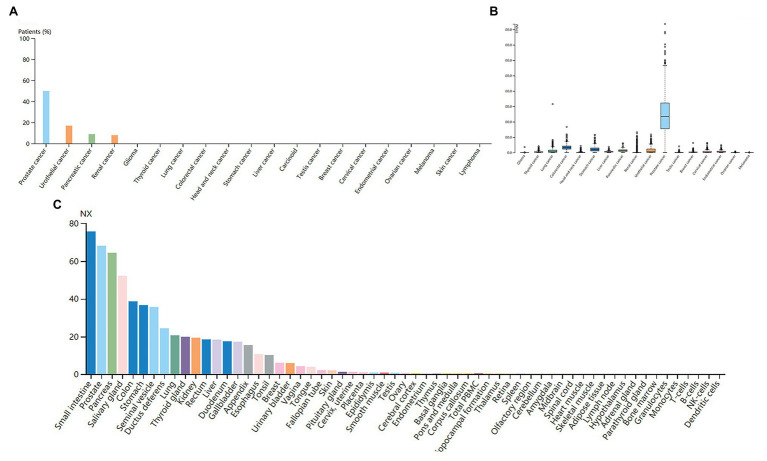
**(A)** Immunoreactivity of TMPRSS2 in various cancers. **(B)** RNA expression of TMPRSS2 in different cancers in the HAC database. Prostate cancer obviously enriched **(C)** RNA tissue specificity: intestine, pancreas, and prostate tissue enhancement.

The high expression level of TMPRSS2 in the tumor and corresponding normal tissues in cancers was verified *via* the Oncomine database. As shown in Figure 1C, TMPRSS2 displayed a higher expression level in bladder cancer, kidney cancer, liver cancer, and prostate cancer, and a lower expression level was found in most other cancers. The prostatic-related microarray GSE6956 contains gene expression profiles of primary prostate tumors resected from 69 patients and 18 non-tumor prostate tissues. The Wilcoxon test and *t*-test were used to compare the expressions of tumor and normal groups of data. The results (Figure 1A) showed that the expression of TMPRSS2 in prostate cancer tissues was significantly higher than that in normal tissues (value of *p* < 0.001) GES6956 further proves the higher result in prostate cancer compared with normal tissue. The RNA-seq expression data in tumors from the TCGA TIMER database (Figure 1D) show that TMPRSS2 displays obviously high expression in PRAD. From the GTEx projects, we know that the TMPRSS2 gene is highly expressed in the prostate (Figure 1B). We also explored the expression of TMPRSS2 between tumor and normal tissues and conducted IHC in the HPA database. The protein expression of TMPRSS2 was significantly reduced in normal tissue, and the protein level was significantly elevated in tumor tissue (Figures 2A–D). As shown in Figure 3A, prostate cancer, selected renal cell cancers, urothelial carcinoma, lung cancer, colorectal cancer, and pancreatic cancer exhibit weak to moderate membranous or granular cytoplasmic immunoreactivity. The remaining cancer tissues were all negative. Based on the The Cancer GenomeAtlas (TCGA) database, the gene was enriched in prostate cancer in the HPA database (Figure 3B), and RNA tissue specificity was similarly enriched in prostate cancer (Figure 3C). The higher expression level suggests that TMPRSS2 possesses diverse functions in various tumors, especially in PRAD.

### TMPRSS2 Prognostic Value With PRAD

**Figure 4:**
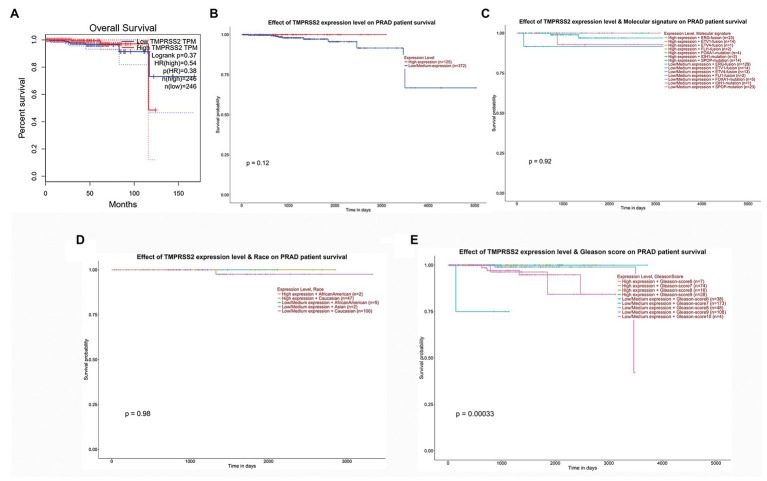
Correlation between TMPRSS2 and patient survival. **(A)** Overall survival (OS) of PRAD (*p* < 0.5) on gene expression profiling interactive analysis (GEPIA), **(B)** effect of TMPRSS expression on PRAD patient survival, **(C)** effect of molecular signature on PRAD patient survival, **(D)** effect of race on PRAD patient survival, and **(E)** effect of Gleason score on PRAD patient survival.

We used the GEPIA database to examine the prognostic value of TMPRSS2. We calculated the Cox P/log-rank *p* value and hazard ratio with 95% intervals. We set Cox P/log-rank *p* = 0.05 as the thresholds. The patients were divided into two groups based on the median level of TMPRSS2 expression in each queue. Univariate analysis was performed through GEPIA to assess the impact of TMPRSS2 on various cancer survival rates (Figure 4A). The results showed that the expression level of TMPRSS2 had an effect on the prognosis of PRAD. Moreover, the UALCAN database was used to evaluate the effect of TMPRSS expression, molecular signature, race and Gleason score on PRAD patient survival. The results showed that prostate adenocarcinoma patients with a high level of TMPRSS2 expression, and Gleason score exhibited a longer survival period (Figure 4E). This result means that patient survival is associated with gene expression and Gleason score rather than molecular signature and race (Figures 4B–E). Given this information, these results suggested that high expression of TMPRSS2 was related to good prognosis of PRAD.

### TMPRSS2 Immune Regulation Molecules

**Figure 5:**
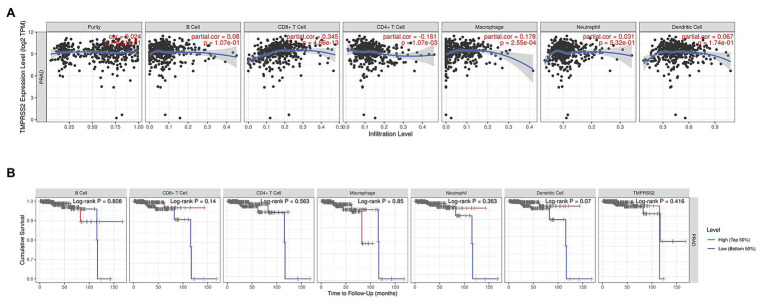
Correlation between TMPRSS2 expression and immune infiltration in PRAD in tumor immune assessment resource (TIMER) database. **(A)** Correlation between TMPRSS2 expression and immune cell infiltration in PRAD. CD8^+^ T-cells, CD4^+^ T-cells, and macrophages relative to TMPRSS2 expression (*p* < 0.001). **(B)** Survival analysis between TMPRSS2 expression and immune cells in PRAD. CD8^+^ T-cell, neutrophil, and dendritic cell were connected with high expression and good prognostic value.

The higher TMPRSS2 expression level suggests that it possesses diverse functions in various tumors, and we explored its GO function in a GO model of TISIDB database, where we found TMPRSS involved in multiple functions related to virus entry into the host cell and viral life cycle. The important role of TMPRSS in regulating the virus suggests its potential association with immune cells in the tumor microenvironment. *Via* GO and KEGG analyses of GSE30589 and GSE52920 in R, TMPRSS2 was further found to be involved in a variety of virus-related functions (Table 1) and multiple immune-related pathways (Table 2). To explore whether TMPRSS2 exerts potential biological roles in immune infiltration, we conducted an integrated analysis based on the TIMER and TISIDB databases, analyzing the link between TMPRSS2 and immune cell infiltration as well as gene markers of immune cell subtypes in PRAD. As the consequence in Figure 5, it suggested that high levels of TMPRSS2 mRNA expression were associated with high immune infiltration in PRAD. The TMPRSS2 mRNA expression level was significantly negatively correlated with infiltrating levels of CD8^+^ T-cells (*r* = −0.345, *p* = 4.66*e* ^−13^) and CD8^+^ T-cells (*r* = −0.16, *p* = 1.07*e* ^−003^), and it was positively correlated with macrophages (*r* = 0.178, *p* = 2.55*e* ^−04^; Figure 5A).

**Table 1:** TMPRSS2 participated in the GO function analysis results of GSE30589 and GSE56677.

ID	Description	*p* value	*p* adjust	*q* value	Count
GO:0019058	Viral life cycle	2.05E-11	1.78652E-09	1.5001E-09	603
GO:0051604	Protein maturation	0.000339827	0.004809938	0.00424586	490
GO:0043903	Regulation of symbiosis, encompassing mutualism through parasitism	3.53946E-07	9.66637E-06	8.1236E-06	395
GO:0044409	Entry into host	1.65956E-06	4.46046E-05	3.8172E-05	129
GO:0046718	Viral entry into host cell	3.09906E-06	7.24812E-05	6.0859E-05	334
GO:0050792	Regulation of viral process	4.0981E-06	8.06443E-05	6.9626E-05	195
GO:1903902	Positive regulation of viral life cycle	8.46858E-05	0.001138812	0.0009563	138
GO:1903900	Regulation of viral life cycle	0.000104432	0.001346311	0.00113051	267
GO:0060090	Molecular adaptor activity	5.41512E-07	4.84927E-05	4.6298E-05	361
GO:0051701	Interaction with host	2.86073E-13	5.25868E-11	4.642E-11	179
GO:0048524	Positive regulation of viral process	2.23649E-08	1.2496E-06	1.1031E-06	96
GO:0046596	Regulation of viral entry into host cell	7.83088E-05	0.001557809	0.00137512	29
GO:0052372	Modulation by symbiont of entry into host	0.000330315	0.005246778	0.00463147	34

**Table 2:** TMPRSS2 participated in the KEGG enrichment analysis results of GSE30589 and GSE56677.

ID	Description	*p* value	*p* adjust	*q* value	Count
hsa04144	Endocytosis	5.55929E-05	0.000392977	0.00016009	235
hsa05202	Transcriptional misregulation in cancer	0.048582347	0.07965532	0.0324488	170
hsa04115	p53 signaling pathway	5.80884E-06	5.21159E-05	2.7854E-05	67
hsa04210	Apoptosis	7.1629E-06	5.78404E-05	3.0914E-05	119
hsa05202	Transcriptional misregulation in cancer	0.00817856	0.022971086	0.0122772	148
hsa04390	Hippo signaling pathway	0.013933685	0.036295002	0.01939834	125
hsa04215	Apoptosis-multiple species	0.074979651	0.147673337	0.07892595	27

**Figure 6:**
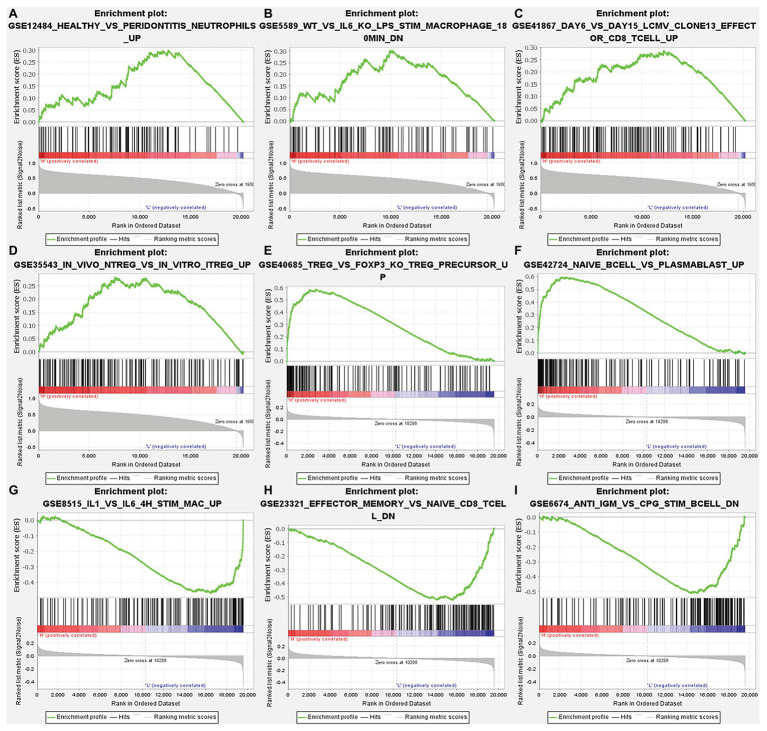
Analysis of TMPRSS2 single-gene gene set enrichment analysis (GSEA) based on C7: immunologic gene sets in GSE30589 and GSE5667. **(A)** GSE12484 healthy vs. peridontitis neutrophils up. **(B)** GSE5589 wt vs. il6 ko lps stim macrophage 180 min dn. **(C)** GSE41867 day 6 vs. day 15 lcmv clone13 effect or CD8 T-cell up. **(D)** GSE35543 *in vivo* ntreg vs. *in vitro* itreg up. **(E)** GSE40685 trfg vs. foxp3 ko treg precursor up. **(F)** GSE42724 naïve bcell vs. plasmablast up. **(G)** GSE8515 il1 vs. il6 4 h stim mac up. **(H)** GSE23321 effector memory vs. naïve cd8 t cell dn. **(I)** GSE6674 anti igm vs. cpg stim B cell dn.

**Figure 7:**
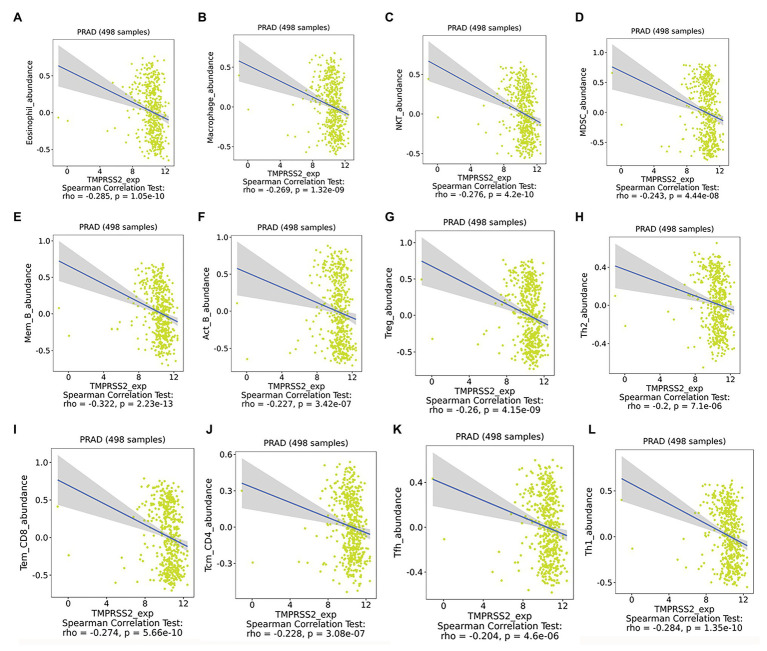
Expression of TMPRSS2 was associated with tumor-infiltrating lymphocytes (TILs). **(A)** Eosinophil_abundance, **(B)** Macrophase_abundance, **(C)** NKT_abundance, **(D)** MDSC_abundance, **(E)** Mem_B_abundance, **(F)** Act_B_abundance, **(G)** Treg_abundance, **(H)** Th2_abundance, **(I)** Tem_CD8_abundance, **(J)** Tcm_CD4_abundance, **(K)** Tfh_abundance, and **(L)** Th1_abundance.

**Figure 8:**
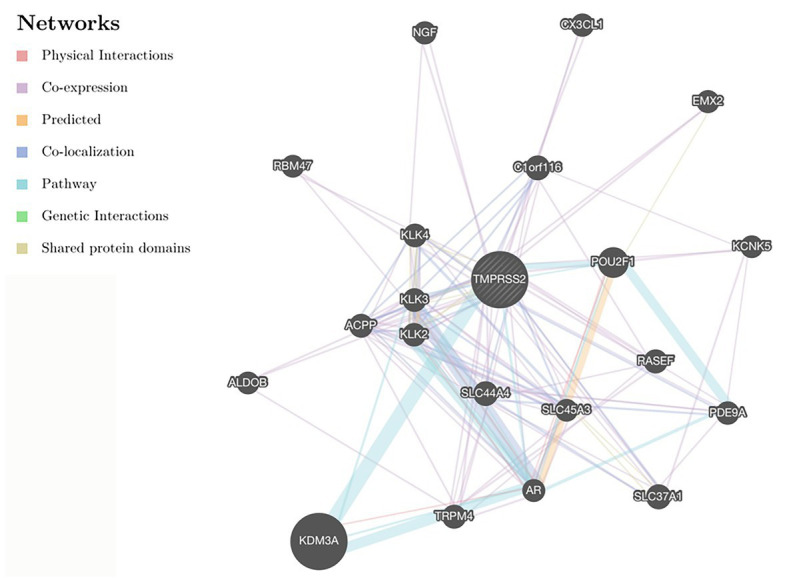
Protein-protein interaction (PPI) network in GENEMANIA database.

After correcting the tumor purity, the immune cell type markers in PRAD were further studied. Table 3 also shows that the TMPRSS2 mRNA expression level had significant correlations with B-cells (CD19, CD27, and CD38), CD8^+^ T-cells (CD8A and CD8B), neutrophils (FCGR3B, SIGLEC5, and S100A12), macrophages (CD84 and CD163), Th1 (STAT4 and STAT1), Treg (STAT5B and TGFB1), and T-cell exhaustion (PDCD1, CTLA4, LAG3, and GZMB) in PRAD. Through single-gene GSEA analysis based on “C7: Immunologic Gene Sets,” the biological role of TMPRSS2 in the tumor environment was more specifically reflected, as shown in Figure 6. These results strongly confirmed the correlation between TMPRSS2 and immune infiltration in PRAD. In a further investigation, we found that the expression of TMPRSS2 was associated with tumor-infiltrating lymphocytes (TILs), including activated eosinophil, macrophage, natural killer T-cell, myeloid derived suppressor cell, memory B-cell, active B-cell, regulatory T-cell, type-2 helper cell, effector memory CD8 T-cell, central memory CD4 T-cell, T follicular helper cell, and Type-1 T helper cell (Figures 7A–L). The *p* values of all of the abovementioned cells are less than 0.001, and |rho| ≥ 0.2. Besides, we also explored the biological network between TMPRSS2 and PRAD as shown in Figure 8. Overall, these results suggested that TMPRSS2 and its associated genes were important for immune cell infiltration in the PRAD microenvironment and possibly have a more significant effect on the prognosis of PRAD.

**Table 3:** Correlation analysis between PRAD and related gene markers of immune cells.

Cell type	Gene markers	PRAD					
		None		Purity		Age	
		Cor	*p* value	Cor	*p* value	Cor	*p* value
B Cell	CD19	−0.125077723	^**^	−0.161441404	^***^	−0.130674148	^**^
	CD27	−0.148507385	^***^	−0.197633538	^***^	−0.147497971	^**^
	CD38	0.277349561	^***^	0.264768159	^***^	0.285518628	^***^
	CD40	−0.031102373	0.488490749	−0.065324277	0.182988272	−0.014884155	0.743096953
	CD80	−0.025275706	0.573623548	−0.000775715	0.987399525	−0.028181983	0.5349634
	CXCR3	−0.058626234	0.191438313	−0.092155904	0.060097398	−0.050598292	0.264982434
	CXCR4	−0.039409599	0.213990606	−0.060386012	0.081356974	−0.039569694	0.217267393
	CXCR5	−0.111933944	^*^	−0.147159268	^**^	−0.11526283	^*^
	CXCR6	0.04599172	0.305688784	0.004643028	0.92465759	0.054955353	0.226065083
	CD24	−0.026473203	0.555471904	−0.015678495	0.749457539	−0.022785709	0.615822823
	NOTCH2	0.385737482	^***^	0.412167843	^***^	0.38647116	^***^
	TLR4	0.058113222	0.195428891	0.037421509	0.44597527	0.07310733	0.107103676
	FCRL2	−0.0689383	0.124444368	−0.09415165	0.054715415	−0.06900531	0.128331543
	MS4A1	−0.050275534	0.262784776	−0.09070561	0.064239045	−0.051592392	0.255799248
CD8^+^ T cells	CD8A	−0.014071002	0.754020343	−0.057182925	0.243844749	−0.003878102	0.931948835
	CD8B	−0.253819947	^***^	−0.294883269	^***^	−0.239926201	^***^
Neutrophils	FCGR3B	0.227748682	^***^	0.192310891	^***^	0.239183779	^***^
	CEACAM3	−0.084244806	0.060296919	−0.124806186	^*^	−0.074381723	0.10110602
	SIGLEC5	0.127578293	^**^	0.108113169	^*^	0.137689965	^**^
	FPR1	0.017907041	0.69005842	0.005304498	0.913962729	0.018479869	0.684049454
	CSF3R	0.013799921	0.758610788	−0.007289568	0.881968209	0.024218738	0.593795613
	S100A12	0.101840483	^*^	0.050768182	0.301009622	0.103858731	^*^
	CCR7	−0.055605383	0.0794254	−0.095131944	^**^	−0.056713265	0.07687487
	CD59	0.051576816	0.103784756	−0.000217208	0.995002609	0.069817898	^*^
	ITGAM	0.01456332	0.646192653	−0.012419306	0.72024322	0.025774571	0.421686702
Macrophages	CD68	0.054701388	0.222928601	0.049576772	0.312372981	0.061720187	0.173819383
	CD84	0.194831995	^***^	0.192929791	^***^	0.201153192	^***^
	CD163	0.114147133	^*^	0.094016855	0.05508935	0.119318014	^**^
	MS4A4A	0.074285586	0.09773774	0.062607569	0.201903638	0.076906158	0.090010272
Th1	STAT4	−0.110499198	^*^	−0.143634834	^**^	−0.102844157	^*^
	TBX21	−0.050166722	0.263820499	−0.079626913	0.104434358	−0.03776681	0.405637131
	CD4	0.010785723	0.810256304	−0.016327057	0.739565125	0.017620344	0.698106353
	STAT1	0.251855636	^***^	0.243150567	^***^	0.262609113	^***^
	IFNG	0.008749306	0.845579407	0.023176929	0.636977475	0.004087266	0.928313694
Th2	GATA3	−0.052853373	0.238972752	−0.076308607	0.119712115	−0.040236079	0.375482123
	CCR4	0.102475485	^*^	0.087114627	0.075573072	0.109502291	^*^
	CCR8	0.083460773	^**^	0.082478046	^*^	0.080220846	^*^
Treg	FOXP3	0.098345298	^*^	0.092100968	0.060230149	0.103534079	^*^
	STAT5B	0.162853597	^***^	0.132678142	^**^	0.174846349	^***^
	TGFB1	−0.121657052	^**^	−0.130643512	^**^	−0.112691622	^*^
Monocyte	CCL2	−0.156503402	^***^	−0.160935808	^***^	−0.154647011	^***^
	IL10	0.102323948	^*^	0.084489676	0.084846693	0.105747627	^*^
	VSIG4	0.055510551	0.216151232	0.037773259	0.441550846	0.069920753	0.123303746
	CSF1R	0.021901893	0.489925764	0.003644287	0.916307499	0.02888442	0.36786306
	FCGR2A	0.04146666	0.355778871	0.043120956	0.379769962	0.046113184	0.309841578
	FCER2	−0.063291987	0.158456007	−0.091735378	0.061259972	−0.06412007	0.157707324
	C3AR1	0.081165021	0.070351302	0.075515738	0.123613844	0.089745028	^*^
	CD86	−0.047062539	0.294553275	−0.068561541	0.162260698	−0.043984262	0.332733556
Dendritic cells	ITGAX	−0.017242456	0.700997541	−0.023215473	0.636282915	−0.011063195	0.807532086
	CD1C	−0.060784439	0.175639379	−0.098711438	^*^	−0.0481207	0.289228125
	NRP1	0.066998266	0.135388895	0.081357421	0.097078769	0.079412634	0.079992779
	THBD	−0.045113816	0.315023163	−0.058283278	0.234878596	−0.034418563	0.448556258
	HLA-DPA1	−0.029792635	0.507121538	−0.057940216	0.237756443	−0.01996682	0.660269707
	CD209	0.13810813	^**^	0.100561136	^*^	0.150573382	^***^
NK cells	KIR3DL3	0.039328973	0.381141308	0.03331388	0.497495954	0.029879363	0.510645775
T Cell exhaustion	PDCD1	−0.157463315	^***^	−0.208662551	^***^	−0.153710866	^***^
	CTLA4	−0.14509021	^**^	−0.162964237	^***^	−0.146369875	^**^
	LAG3	−0.17683449	^***^	−0.211773634	^***^	−0.173234006	^***^
	GZMB	−0.102999687	^*^	−0.120962856	^*^	−0.10075826	^*^

^*^ *p* < 0.05, ^**^ *p* < 0.01, and ^***^ *p* < 0.001.

### Promoter Methylation Levels of TMPRSS2 Decreased in PRAD

**Figure 9:**
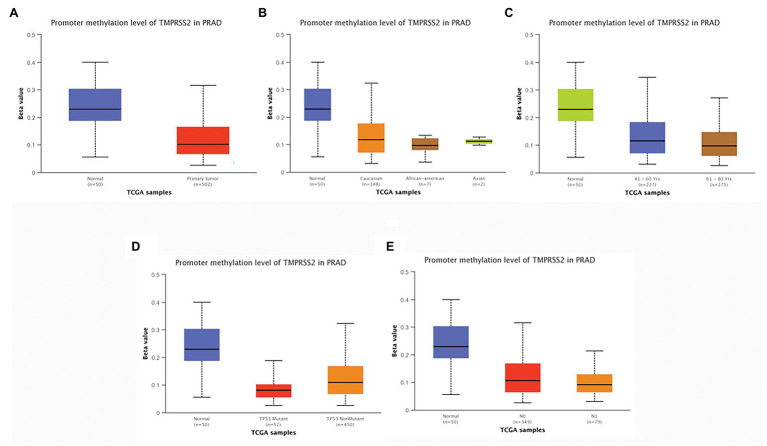
Promoter methylation levels of TMPRSS2 in PRAD. Promoter methylation levels of TMPRSS2 were low in **(A–E)**. PRAD: **(A)** sample type, **(B)** race, **(C)** age, **(D)** lymphatic metastatic status, and **(E)** TP53 mutation status (^*^ *p* < 0.05, ^**^ *p* < 0.01, and ^***^ *p* < 0.001).

**Figure 10:**
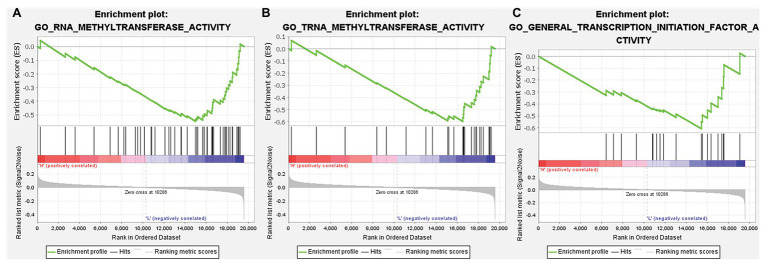
GSEA of gene ontology (GO) gene sets analysis of TMPRSS2 gene in data set GSE30589 and GSE56677. **(A)** RNA methyltransferase activity, **(B)** TRNA methyltransferase activity, and **(C)** general transcription initiation factor activity.

According to the above analysis, we observed a significant increase of TMPRSS2 expression in PRAD, and as a consequence, a further study was performed to explore the reason for the elevated TMPRSS2. Methylation is an important event in epigenetic modification of the genome and is closely related to the course of disease. Particularly, hypomethylation can lead to genome instability (Heyn and Esteller, 2012) and might activate related genes. Therefore, we used the UALCAN database to verify the methylation levels of TMPRSS2 promoter in PRAD. Besides, as shown in the Figures 9B–E, the results is as same as the Figure 9A that the methylation level of TMPRSS2 promoter in normal group was significantly higher than other the groups of race, age, lymphatic metastatic status and TP53 mutation status. The result is shown in Figure 9A. The methylation level of TMPRSS2 promoter in normal tissue was significantly higher than that in PRAD. At the same time, single-gene GSEA analysis of TMPRSS2 was conducted in data sets GSE30589 and GSE52920, and the GSEA of GO gene sets analysis further verified the effect of TMPRSS2 on the promoter methylation level, as shown in Figure 10. Additionally, we performed a stratified analysis of PRAD based on patient race, age, lymphatic metastasis status, and TP53 mutation status, showing that the TMPRSS2 promoter methylation levels of older people, the lymphatic metastasis group, and the TP53 mutation group were lower than that of the control in PRAD (Figures 4B–E), suggesting that PRAD TMPRSS2 promoter methylation might be activated and increase its level.

### SARS-COV-2 Infection Might Increase Expression of TMPRSS2

**Figure 11:**
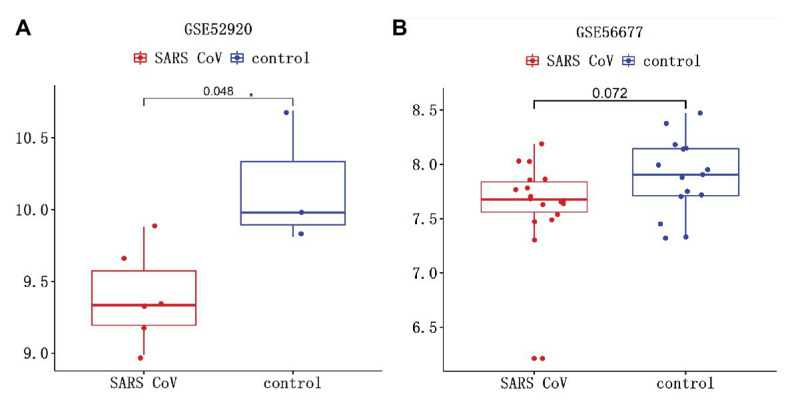
Changes of TMPRSS after severe acute respiratory syndrome-coronavirus (SARS-COV) infection. SARS-COV reduced the expression levels of TMPRSS in **(A)** GSE52920 and **(B)** GSE56677 lungs.

The interaction between TMPRSS2 and ACE2 can promote SARS-COV-2 infection (Hoffmann et al., 2020). The gene TMPRSS2 is closely relevant to prostate cancer as well, regulating many biological processes (Tomlins et al., 2005). Therefore, it is essential to study the variation of TMPRSS2 in PRAD after SARS-COV-2 infection. Clinical features of Middle East respiratory syndrome (MERS) include severe acute pneumonia and renal failure that are highly reminiscent of severe acute respiratory syndrome (SARS) caused by SARS-COV. GSE56677 contained gene expression changes in a human airway epithelial cell line infected with two genetically distinct MERS-COV strains obtained from human patients, MERS-COV-EMC (designated EMC) and MERS-COV-London (designated LoCoV). Triplicate wells of Calu-3 2B4 cells were infected with Human Coronavirus EMC 2012 (HCoV-EMC) or time-matched mock infected. Cells were harvested at 0, 3, 7, 12, 18, and 24 h post-infection (hpi), RNA extracted, and transcriptomics analyzed by microarray (Selinger et al., 2014). Due to the high homology between SARS-COV-2 and SARS-COV, changes in TMPRSS2 expression in cells or animals infected with SARS-COV can be used as a reference for SARS-COV-2 infection (Zhou et al., 2020). GSE52920 contains three biological sample types (SARS-COV-wt, SARS-COV-mutPBM, and Mock) based on mice lung tissue. According to whether the mice infected with SARS-COV, we divided it into two groups, SARS-COV group and Mock group and conducted the analysis on the changes of TMPRSS2 expression between two groups. GSE56677 and GSE52920 were both used to analyze the changes of TMPRSS2 expression in Vero E6 cells and mice lung after SARS-COV infection. The results showed that the expressions of TMPRSS2 in the control group was slightly decreased compared with the other group (Figure 11B), and mice lungs after SARS-COV infection obviously increased compared with the control group (Figure 11A). This finding suggested that TMPRSS2 expression might increase after SARS-COV-2 infection.

**Figure 12:**
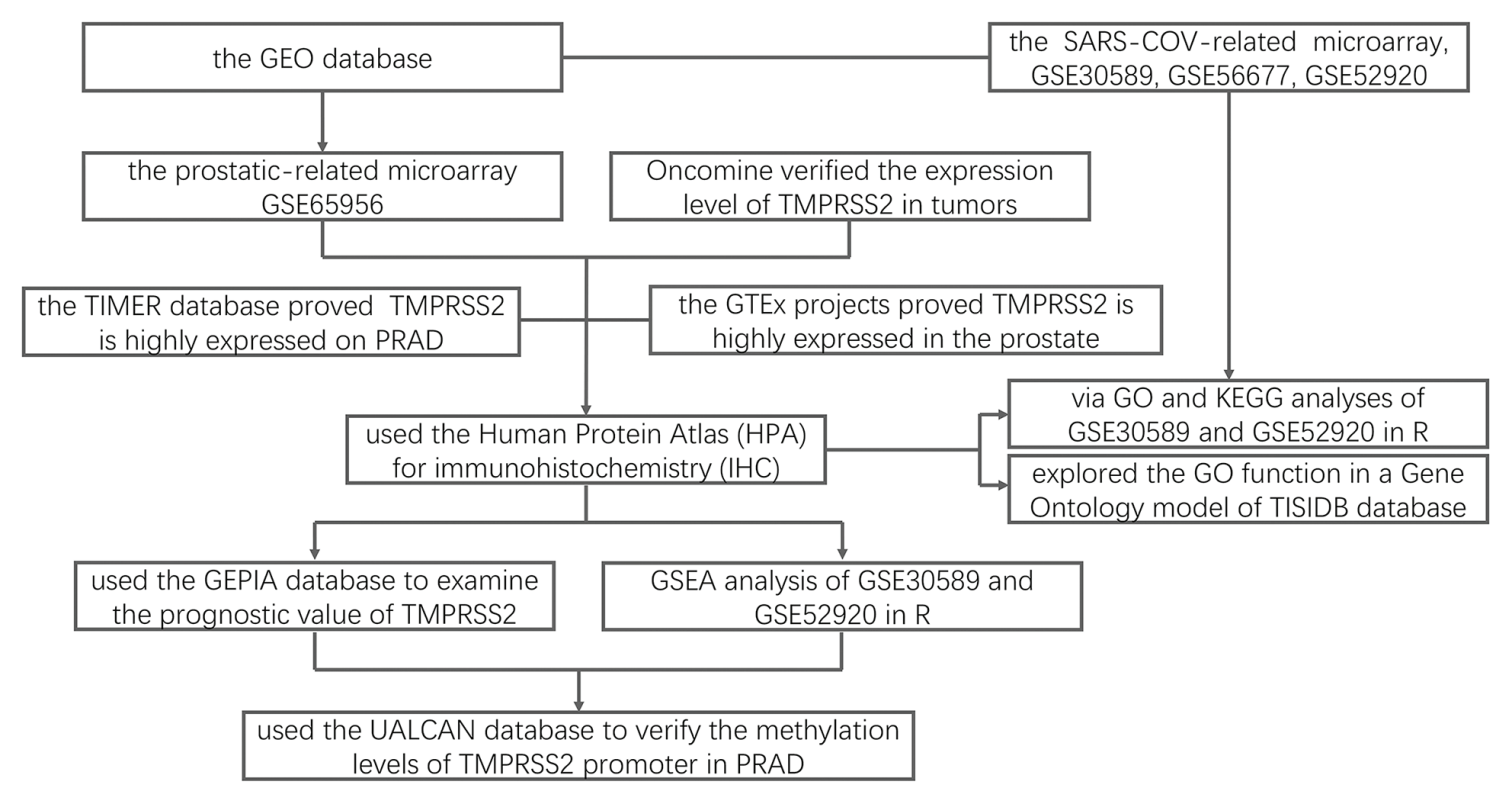
Flowchart for this study. DEGs, differential expression genes; TIMER, tumor immune estimation resource; PRAD, prostatic adenocarcinoma; HPA, Human Protein Atlas; IHC, immunohistochemistry; GO, gene ontology; KEGG, Kyoto encyclopedia of genes and genomes; GSEA, gene set enrichment analysis; and GEPIA, gene expression profiling interactive analysis.

## Discussion

This study analyzed the changes of TMPRSS2 mRNA in PRAD *via* the Oncomine, TIMER and GEO databases and explored the correlation between TMPRSS2 and immune infiltration (Figure 12). In addition, we also respectively investigated the changes before and after TMPRSS2 infection with SARS-COV-2 virus in Vero E6 cells and mouse lungs. The TIMER database based on the TCGA database was used to reveal that TMPRSS2 was also significantly elevated in PRAD (Figure 1D), suggesting that tumor tissues in PRAD were more susceptible to SARS-COV-2 infection. We also used the GERIA and UALCAN databases to process survival analysis and found that the expression of TMPRSS2 was not directly associated with PRAD prognosis. Additionally, the correlation between TMPRSS2 and immune infiltration in PRAD was analyzed in the TIMER database and TISIDB database. The results showed that TMPRSS2 was positively correlated with CD8^+^ T-cells and macrophages in PRAD (Figure 5A). At the same time, single-gene GSEA analysis was used to verify our conclusions. Further studies on immune cell type marker PRAD (Table 1) showed that the expression level of TMPRSS2 mRNA was correlated with B-cells (CD19, CD27, and CD38), and CD8^+^ in PRAD T-cells (CD8A and CD8B), neutrophil granulocytes (FCGR3B, SIGLEC5, and S100A12), macrophages (CD84 and CD163), Th1 (STAT4 and STAT1), Treg (STAT5B and TGFB1), and T-cell failure (PDCD1, CTLA4, LAG3, and GZMB) were significantly correlated, suggesting that these results strongly confirm the close correlation between TMPRSS2 and immune infiltration of PRAD.

To probe the cause of the increased TMPRSS2 in PRAD, we studied the methylation levels of TMPRSS2 in PRAD and found that the promoter methylation levels of TMPRSS2 in LUAD decreased significantly. Hence, TMPRSS2 might be activated and upregulated due to its hypomethylation, explaining the elevated TMPRSS in PRAD to a certain extent. We used GSE56677 and GSE52920 to study the *in vivo* and *in vitro* changes of TMPRSS2 after SARS-COV infection. The consequence shows that TMPRSS2 expression levels in both of GSE56677 and GSE52920 were reduced after SARS-COV infection (Figure 11), suggesting that TMPRSS2 promoter methylation might be activated and display an increased level in PRAD.

As a prostate-specific gene, TMPRSS2 fuses with the transcription factor ERG gene in a large proportion of human prostate cancers (Nam et al., 2007) and plays an important role in selected pathological processes. In certain studies, according to the immunohistochemical analysis of clinical specimens, TMPRSS2 has the highest expression in the apex of the prostate, the secretory epithelium of prostate cancer, and the glandular cavity, indicating that TMPRSS2 is a secreted protease that is highly expressed in prostate cancer and prostate cancer, making it a potential target for the treatment and diagnosis of cancer (Afar et al., 2001). One study showed that considering the high incidence of prostate cancer and the high frequency of such gene fusion, the most common genetic abnormality described thus far in human malignancies is *tmprss2*-ets gene fusion (Rubin and Chinnaiyan, 2006). In addition, TMPRSS2 is a candidate proteolytic activated human influenza virus, which might play an important role in screening other progenitors in the future (Böttcher et al., 2006).

At the same time, studies have found that TMPRSS2 cells are a useful experimental system for studying the cleavage and inhibition of HA by host cell proteases. In addition, these cells also represent a suitable cell line for propagation of the influenza virus in the absence of trypsin (Wu et al., 2017). Interestingly, TMPRSS2 can cleave SARS-COV-2 spike protein, thus facilitating viral entry and activation (Hoffmann et al., 2020), which suggest its correlation with SARS-COV-2. Other studies also show that TMPRSS2-expressing cell lines are highly susceptible to SARS-COV, MERS-COV, and SARS-COV-2 (Matsuyama et al., 2020). In general, TMPRSS2 primarily affects tumor metastasis by intervening in the signaling pathway, but the mechanism of its influence on the prognosis of PRAD is still unclear. However, we found that TMPRSS2 might influence the prognosis of PRAD through a new mechanism, namely, immune infiltration, which suggests a direction for further studies. However, due to the limitations of the database, this study also had certain limitations, and therefore, we did not further analyze the relationship between TMPRSS2 and immune infiltration. Moreover, it is worth noting that all analyses in this paper are based on servers or databases, which may vary in the specific experimental process. In our future research, it will be important to verify the analysis results through experiments.

## Data Availability Statement

All datasets presented in this study are included in the article/supplementary material.

## Author Contributions

LL conceived the idea. LL, YZ, XdL, XnL, and XlL contributed to the acquisition, analysis, and interpretation of data. LL and YZ wrote the manuscript. ML, HL, LC, and LL reviewed the paper and provided comments. All authors reviewed the manuscript. All authors contributed to the article and approved the submitted version.

### Conflict of Interest

The authors declare that the research was conducted in the absence of any commercial or financial relationships that could be construed as a potential conflict of interest.

## Footnotes

^1^http://www.oncomine.org

^2^https://cistrome.shinyapps.io/timer/

^3^http://gepia.cancer-pku.cn/index.html

^4^http://ualcan.path.uab.edu/

^5^http://www.genemania.org

^6^www.proteinatlas.org

^7^http://cis.hku.hk/TISIDB

^8^https://www.ncbi.nlm.nih.gov/geo/

^9^https://www.gtexportal.org/home

^10^http://software.broadinstitute.org/gsea/index.jsp
